# Identification of new channels by systematic analysis of the mitochondrial outer membrane

**DOI:** 10.1083/jcb.201706043

**Published:** 2017-11-06

**Authors:** Vivien Krüger, Thomas Becker, Lars Becker, Malayko Montilla-Martinez, Lars Ellenrieder, F.-Nora Vögtle, Helmut E. Meyer, Michael T. Ryan, Nils Wiedemann, Bettina Warscheid, Nikolaus Pfanner, Richard Wagner, Chris Meisinger

**Affiliations:** 1 Division of Biophysics, School of Biology/Chemistry, University of Osnabrück, Osnabrück, Germany; 2 Institute of Biochemistry and Molecular Biology, ZBMZ, Faculty of Medicine, University of Freiburg, Freiburg, Germany; 3 BIOSS Centre for Biological Signalling Studies, University of Freiburg, Freiburg, Germany; 4 Institute of Biology II, Biochemistry – Functional Proteomics, Faculty of Biology, University of Freiburg, Freiburg, Germany; 5 Leibniz-Institut für Analytische Wissenschaften-ISAS-e.V., Dortmund, Germany; 6 Department of Biochemistry and Molecular Biology, Monash Biomedicine Discovery Institute, Monash University, Melbourne, Australia; 7 Biophysics, Life Sciences and Chemistry, Jacobs University Bremen, Bremen, Germany

## Abstract

The mitochondrial outer membrane is essential for communication between mitochondria and the rest of the cell and facilitates the transport of metabolites, ions, and proteins. All mitochondrial outer membrane channels known to date are β-barrel membrane proteins, including the abundant voltage-dependent anion channel and the cation-preferring protein-conducting channels Tom40, Sam50, and Mdm10. We analyzed outer membrane fractions of yeast mitochondria and identified four new channel activities: two anion-preferring channels and two cation-preferring channels. We characterized the cation-preferring channels at the molecular level. The mitochondrial import component Mim1 forms a channel that is predicted to have an α-helical structure for protein import. The short-chain dehydrogenase-related protein Ayr1 forms an NADPH-regulated channel. We conclude that the mitochondrial outer membrane contains a considerably larger variety of channel-forming proteins than assumed thus far. These findings challenge the traditional view of the outer membrane as an unspecific molecular sieve and indicate a higher degree of selectivity and regulation of metabolite fluxes at the mitochondrial boundary.

## Abstract (precis)

Channels in the mitochondrial outer membrane exchange metabolites, ions, and proteins with the rest of the cell. Kruger et al. identify several new types of channel and suggest that the outer mitochondrial membrane is a more selective molecular sieve with a greater variety of channel-forming proteins than previously appreciated.

## Introduction

Mitochondria fulfill essential functions in cellular energetics, metabolism, and physiology (Neupert and Herrmann, 2007; Youle and van der Bliek, 2012; Labbé et al., 2014; Lill et al., 2014; Wai and Langer, 2016; Braun and Westermann, 2017; Wiedemann and Pfanner, 2017). The outer membrane forms the barrier of mitochondria to the cytosol and is responsible for the transport of a large number of metabolites, ions, and precursor proteins (Neupert and Herrmann, 2007; Endo and Yamano, 2010; Hiller et al., 2010; Peixoto et al., 2010; Schmidt et al., 2010; Harsman et al., 2011; Hewitt et al., 2011; Colombini, 2012). Only a small number of channel-forming outer membrane proteins are known, all of which are β-barrel proteins. This includes the abundant voltage-dependent anion channel (VDAC), also termed Porin. VDAC is present in two (yeast) or three (human) isoforms and mediates the flux of metabolites and ions into and out of mitochondria (Benz, 1990; Hiller et al., 2010; Palmieri and Pierri, 2010; Colombini, 2012; Mertins et al., 2014; Naghdi and Hajnóczky, 2016). The other known outer membrane channels (OMCs) transport precursor proteins including the main protein import channel Tom40 of the translocase of outer mitochondrial membrane complex, the channel Sam50 of the sorting and assembly machinery, and the mitochondrial distribution and morphology protein Mdm10. Electrophysiological analyses revealed a cation preference of Tom40, Sam50, and Mdm10 (Hill et al., 1998; Künkele et al., 1998; Ahting et al., 1999; Paschen et al., 2003; Suzuki et al., 2004; Becker et al., 2005; Bredemeier et al., 2007; Kutik et al., 2008a; Poynor et al., 2008; Ellenrieder et al., 2016). Tom40 translocates several hundred different precursor proteins with positively charged targeting signals from the cytosol into mitochondria, whereas Sam50 inserts β-barrel precursors into the outer membrane (Neupert and Herrmann, 2007; Endo and Yamano, 2010; Schmidt et al., 2010). Mdm10 has a dual role: it associates with the sorting and assembly machinery to promote biogenesis of the translocase of outer mitochondrial membrane and functions as a membrane anchor of the ER–mitochondria encounter structure (Meisinger et al., 2004; Kornmann et al., 2009; Wideman et al., 2010; Becker et al., 2011a; Klein et al., 2012; Ellenrieder et al., 2016). VDAC, Tom40, and Mdm10 form a family of 19-stranded mitochondrial β-barrel proteins. Sam50 has been conserved from the prokaryotic Omp85/BamA family of 16-stranded β-barrel proteins.

Several studies indicate that VDAC is not just a constitutively open pore but that its activity can be regulated (Vander Heiden et al., 2000; Azoulay-Zohar et al., 2004; Rostovtseva et al., 2008; Arbel and Shoshan-Barmatz, 2010; Herrmann and Riemer, 2010; Colombini, 2012; Lemasters et al., 2012; Maldonado et al., 2013; Mertins et al., 2014; Naghdi and Hajnóczky, 2016; Campo et al., 2017). Reconstituted VDAC shows a preference for anions at high conductance states and for cations at low conductance states (Benz, 1990; Colombini, 2012; Mertins et al., 2014; Campo et al., 2017). Distinct functions have been assigned to the three VDAC isoforms in mammals (Cheng et al., 2003; Baines et al., 2007; Colombini, 2012; Plötz et al., 2012; Mertins et al., 2014; Naghdi and Hajnóczky, 2016). The deletion of both VDAC isoforms Por1 and Por2 is not lethal in yeast but causes growth defects (Blachly-Dyson et al., 1997). It was suggested that in the absence of VDAC, the Tom40 channel may function in the translocation of crucial metabolites (Kmita and Budzińska, 2000; Antos et al., 2001). Alternatively, it is conceivable that further channels may exist in the mitochondrial outer membrane. Outer membrane transporters for porphyrin and cholesterol were found in mammalian mitochondria, but no channel activity was observed (Krishnamurthy et al., 2006; Rupprecht et al., 2010; Fan and Papadopoulos, 2013; Li et al., 2015). Based on the limited set of known OMCs, however, the traditional model of a molecular sieve with rather large unspecific pores has remained one of the views of the mitochondrial outer membrane.

The number of mitochondrial β-barrel proteins is limited. Systematic analyses of the whole genome indicated that with VDAC, Tom40, Sam50, and Mdm10, all β-barrel proteins have been identified (Imai et al., 2008; Kutik et al., 2008b; Zeth, 2010). Our search for putative further channels of the mitochondrial outer membrane included the hypothesis that also non–β-barrel channels may exist. We screened for OMC-forming proteins of yeast mitochondria and discovered four previously unrecognized channel activities. This includes a channel formed by the mitochondrial import (MIM) complex that is anchored in the outer membrane via predicted α-helical transmembrane segments (Popov-Čeleketić et al., 2008) as well as a NADPH-regulated channel formed by a protein previously reported to function in lipid metabolism, 1-acyldihydroxyacetone-phosphate reductase (Ayr1; Athenstaedt and Daum, 2000; Han et al., 2002; Ploier et al., 2013). These results implicate a change in the traditional view of the functional organization of the mitochondrial outer membrane. The number of different channels is considerably higher than expected, suggesting a larger variety of specific outer membrane transport processes and a higher degree of regulation of solute fluxes at the level of the outer membrane.

## Results and discussion

### Search for new channels of the mitochondrial outer membrane

**Figure 1.**
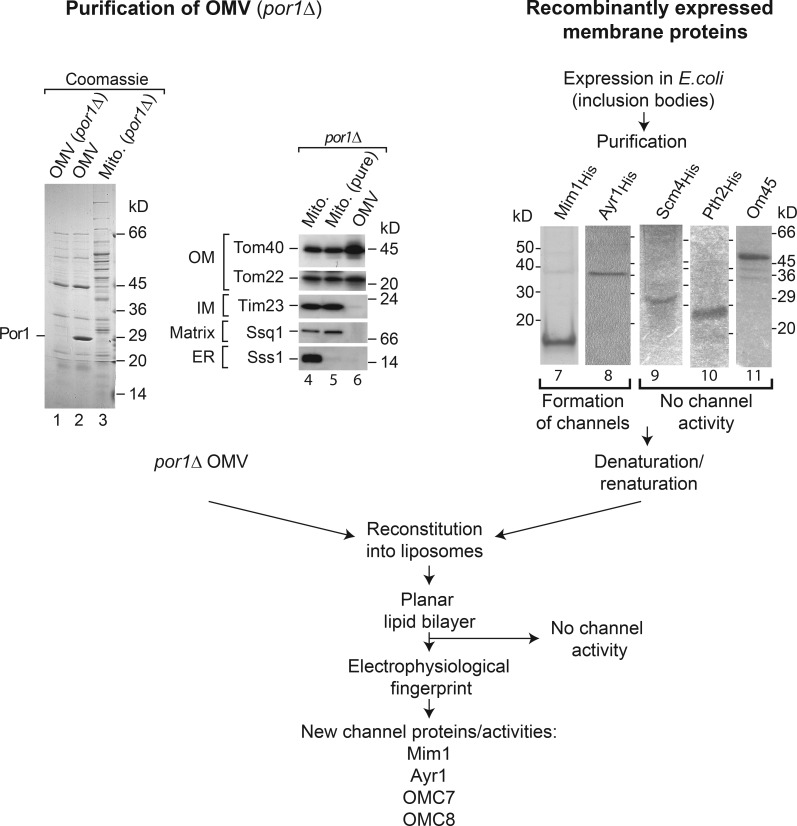
**Screen for channels linked to the yeast mitochondrial outer membrane.** (Lanes 1–3) Mitochondrial outer membrane (OM) vesicles (OMVs) from *por1*Δ and WT yeast and mitochondria (Mito.) were analyzed by SDS-PAGE and Coomassie staining. (Lanes 4–6) Crude and purified mitochondria and outer membrane vesicles from *por1*Δ were analyzed by SDS-PAGE and immunodetection. (Lanes 7–11) Proteins were recombinantly expressed, purified, and analyzed by SDS-PAGE and Coomassie staining. IM, inner membrane.

We used several approaches to characterize mitochondrial OMCs in the model organism baker’s yeast (Fig. 1). The four known β-barrel channels are Por1, Tom40, Sam50, and Mdm10 (Table 1). The second VDAC isoform, Por2, does not measurably contribute to channel activities of the outer membrane; it is only present in tiny amounts compared with Por1 (Kulak et al., 2014; Morgenstern et al., 2017), and its deletion does not affect the outer membrane permeability (Lee et al., 1998). We isolated outer membrane vesicles from purified *por1*Δ yeast mitochondria (Fig. 1, lanes 1–6). Upon reconstitution into liposomes as outlined in the Cellular fractionation, purification, and reconstitution... section of Materials and methods, electrophysiological analysis was performed with planar lipid bilayers. To identify channels on the molecular level, we considered proteins that were found in outer membrane vesicles (Zahedi et al., 2006) and contained at least one predicted transmembrane segment as potential candidates. We selected nine proteins for further analysis, excluding the known β-barrel proteins. Five proteins were efficiently expressed in *Escherichia coli* cells, purified, and reconstituted into liposomes for electrophysiological analysis (Fig. 1, lanes 7–11).

**Table 1.** Channels linked to the yeast mitochondrial outer membrane

**Channel**	**Conductance**	**V_rev_ (mV)**	**P_K_+/P_Cl_-**	**Reference**
Por1	4.5 nS^a^	−11^a^	0.58	Forte et al., 1987
Tom40	360 pS^b^	40^c^	8–10	Hill et al., 1998; Becker et al., 2005
Sam50	640 pS^b^	30^c^	4.5	Kutik et al., 2008a
Mdm10	480 pS^b^	21.5^c^	2.8	Ellenrieder et al., 2016
Mim1	580 pS^b^	53^c^	23.5	This study
Ayr1	1.47 nS^b^	30–43^c^	4.5–10	This study
OMC7	570 pS^b^	−12.5^c^	0.55	This study
OMC8	550 pS^b^	−15.5^c^	0.48	This study

The channels are listed with maximal conductance, reversal potential (V_rev_), and cation/anion selectivity (P_K_+/P_Cl_-).

a Gradient 1 M/100 mM KCl.

b Symmetrical buffer conditions including 250 mM KCl (cis/trans).

c Gradient 250 mM/20 mM KCl (cis/trans).

We identified four new channel activities by their specific electrophysiological fingerprint: the cation-preferring channels Mim1 and Ayr1 as well as the slightly anion-selective OMC7 and OMC8 (Table 1). The channels are distinguishable from each other and the known β-barrel channels by their electrophysiological characteristics, including conductance, selectivity, gating behavior, and specific response to effectors.

**Figure 2.**
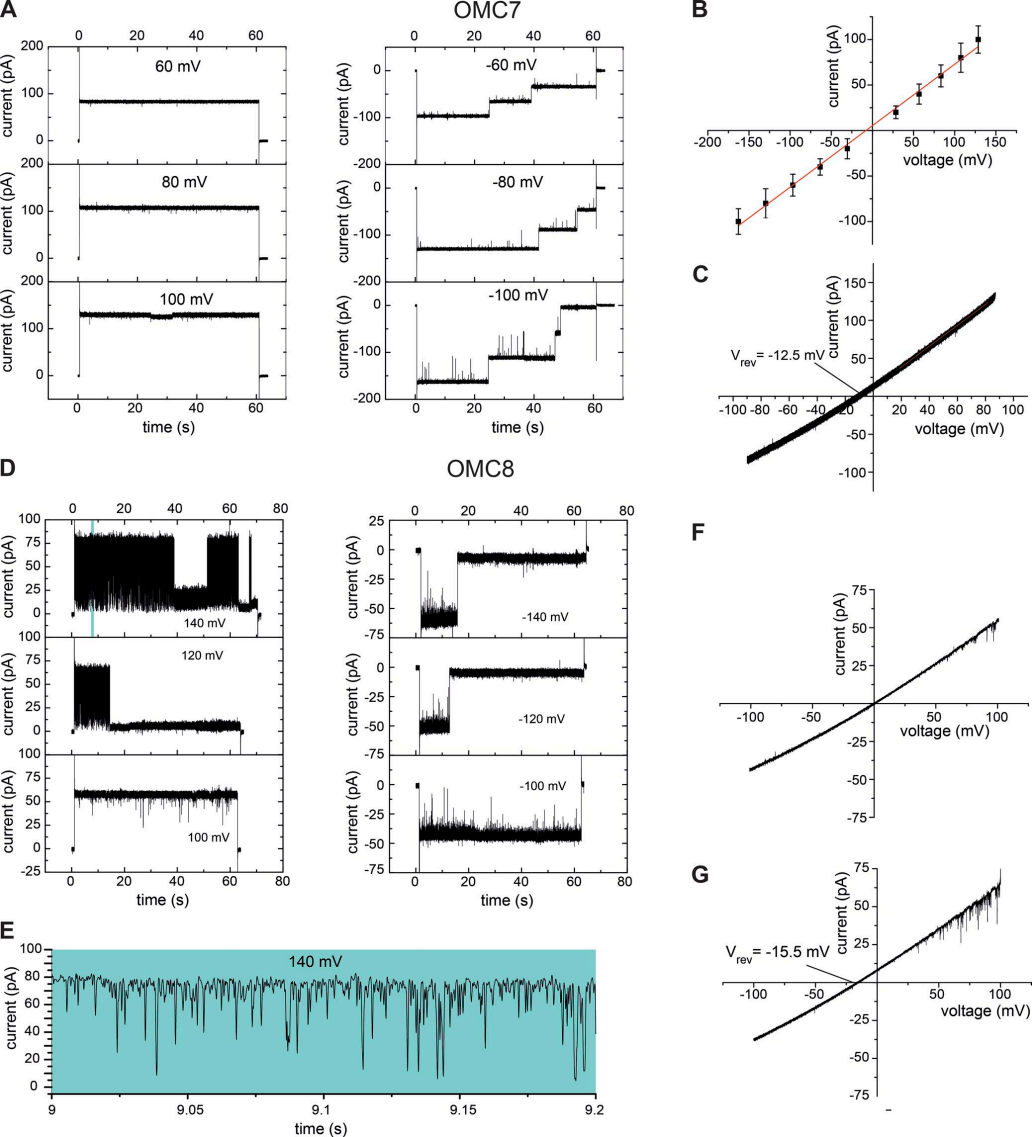
**Characterization of the channel activities OMC7 and OMC8.** (A) Current recordings of the OMC7 channel reconstituted into liposomes in symmetrical (250 mM KCl cis/trans) buffer conditions at the indicated voltage amplitude (V_m_). (B) Current-voltage recordings of OMC7 in symmetrical buffer conditions. Mean values of three independent experiments with SD. (C) Current-voltage recordings of OMC7 in asymmetric (250 mM/20 mM KCl cis/trans) buffer conditions. (D) Current recordings of the OMC8 channel reconstituted into liposomes in symmetrical buffer conditions. (E) Expanded current recording from the top left panel in D. (F) Current-voltage recordings of OMC8 in symmetrical buffer conditions. (G) Current-voltage recordings of OMC8 in asymmetric buffer conditions.

OMC7 and OMC8 were not identified on a molecular level but were defined by their specific electrophysiological properties. OMC7 showed a main conductance transition of ${\bar{\Delta G}}_{main}≅570\;pS$ (Fig. 2, A and B; and Fig. S1 A). OMC7 closed in three single steps in a voltage-dependent manner but closed remarkably faster at negative voltages (−V_m_ ≥ 60 mV; Fig. 2 A). Using asymmetric buffer concentrations, we determined a reversal potential V_rev_ = −12.5 mV (Figs. 2 C and S1 B) corresponding with a moderate anion selectivity of $P_{K^{+}}/P_{Cl^{-}}=0.55$ according to the Goldman-Hodgkin-Katz approach (Hille, 2001).

OMC8 showed a single channel current with a main conductance transition of ${\bar{\Delta G}}_{main}=550\;pS$ and a slightly rectifying current-voltage relation (Fig. 2, D–F; and Fig. S1 A). We determined a reversal potential V_rev_ = −15.5 mV (Figs. 2 G and S1 B) corresponding with an anion selectivity of $P_{K^{+}}/P_{Cl^{-}}=0.48$. A typical fingerprint of the OMC8 activity is its high gating frequency (flickering) at V_m_ > 100 mV (Fig. 2, D and E).

### Mim1 forms a Mim2-sensitive channel

**Figure 3.**
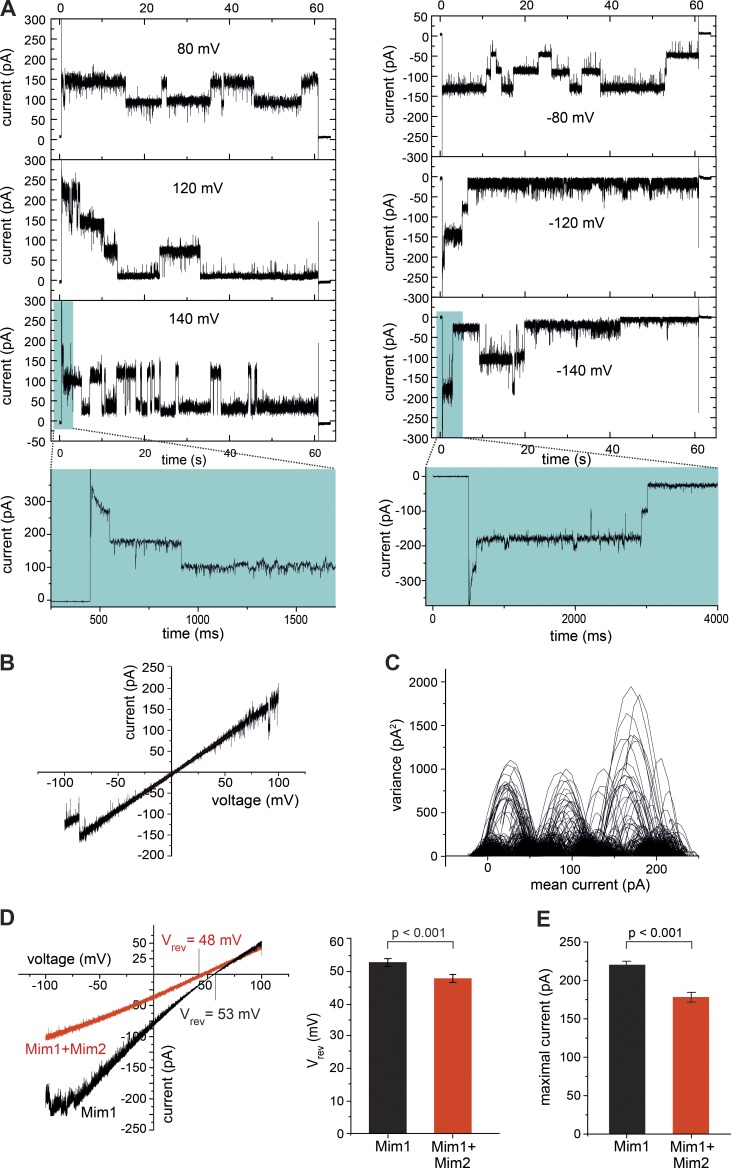
**Mim1 forms a channel.** (A) Current recordings in symmetrical buffer conditions from a bilayer containing active Mim1 channels at the indicated voltage (V_m_). (B) Current-voltage recordings of the Mim1 channel in symmetrical buffer conditions. (C) Mean variance plot of a single Mim1 channel unit calculated from the symmetrical current recording (A) at V_m_ = 120 mV. (D) Current-voltage recordings of the Mim1 channel without (black) or after coreconstitution with Mim2 (red). Mean values of V_rev_ of five (Mim1) and four (Mim1 + Mim2) independent experiments with SD (unpaired *t* test). (E) Mean of the maximal current of the Mim1 channel in the absence (*n* = 15) and presence (*n* = 11) of Mim2.

Mim1 promotes the biogenesis of several α-helical precursor proteins of the mitochondrial outer membrane by an unknown mechanism (Becker et al., 2008, 2011b; Hulett et al., 2008; Popov-Čeleketić et al., 2008; Papić et al., 2011; Dimmer et al., 2012). The 13-kD protein Mim1 contains a predicted central α-helical transmembrane segment and exposes its N-terminal domain to the cytosol and its C-terminal domain to the intermembrane space (Popov-Čeleketić et al., 2008; Lueder and Lithgow, 2009). We expressed full-length Mim1 in *E. coli* cells, where it accumulated in inclusion bodies. Using an N-terminal His tag, Mim1 was purified under denaturing conditions (Fig. 1, lane 7). Upon reconstitution into planar lipid bilayers, Mim1 exhibited a characteristic channel activity that was inhibited by Mim1-specific antibodies (Fig. 3, A and B; and Fig. S2, A–D). Mim1 synthesized in wheat germ lysate exhibited the same channel activity. We observed a main conductance state of ${\bar{\Delta G}}_{main}=580\;pS$ that closed upon application of high positive or negative voltages (Fig. 3 A and Table 1). Current-voltage recordings under symmetrical buffer conditions revealed a slightly rectifying behavior of the Mim1 channel (Fig. 3 B). The Mim1 channel activity typically occurred within three to four multiples of the single channel unit after fusion of Mim1-proteoliposomes with the planar lipid bilayer (Fig. 3 C). The Mim1 channel was cation selective with a ratio of *P_K_^+^/P_Cl_^−^* of 23.5:1 based on a positive reversal potential of 53 mV (Fig. 3 D, Fig. S2 E, and Table 1).

Mim1 oligomers form the major constituent of the MIM complex (Ishikawa et al., 2004; Popov-Čeleketić et al., 2008; Becker et al., 2011b; Dimmer et al., 2012). A second subunit, the 10-kD protein Mim2, possesses the same topology as Mim1 yet is present in the MIM complex in lower abundance (one to two copies per complex; Dimmer et al., 2012). We expressed Mim2 and coreconstituted it with Mim1 in planar lipid bilayers. Mim1–Mim2 exhibited a diminished reversal potential (V_rev_ = 48 mV), leading to a reduced but still high cation selectivity of *P_K_^+^/P_Cl_^−^* = 11:1 (Figs. 3 D and S2 E). The maximal current of reconstituted Mim1–Mim2 was moderately reduced in comparison with reconstituted Mim1 alone (Figs. 3 E and S2 F). Thus, coreconstitution of Mim2 with Mim1 attenuated the channel properties of Mim1 but still retained a considerable cation preference. The cation selectivity of Mim1–Mim2 is higher than that of Tom40 (*P_K_^+^/P_Cl_^−^* ≅ 8:1), which translocates positively charged presequences (Hill et al., 1998; Becker et al., 2005). The substrates of the Mim1–Mim2 complex contain transmembrane segments that are typically flanked by positively charged amino acid residues (Endo and Yamano, 2010; Dukanovic and Rapaport, 2011; Ellenrieder et al., 2015). We conclude that the Mim1–Mim2 complex forms a channel with cation preference and is thus conducive to the translocation of precursor segments carrying positive charges.

### Ayr1 forms an NADPH-regulated channel

**Figure 4.**
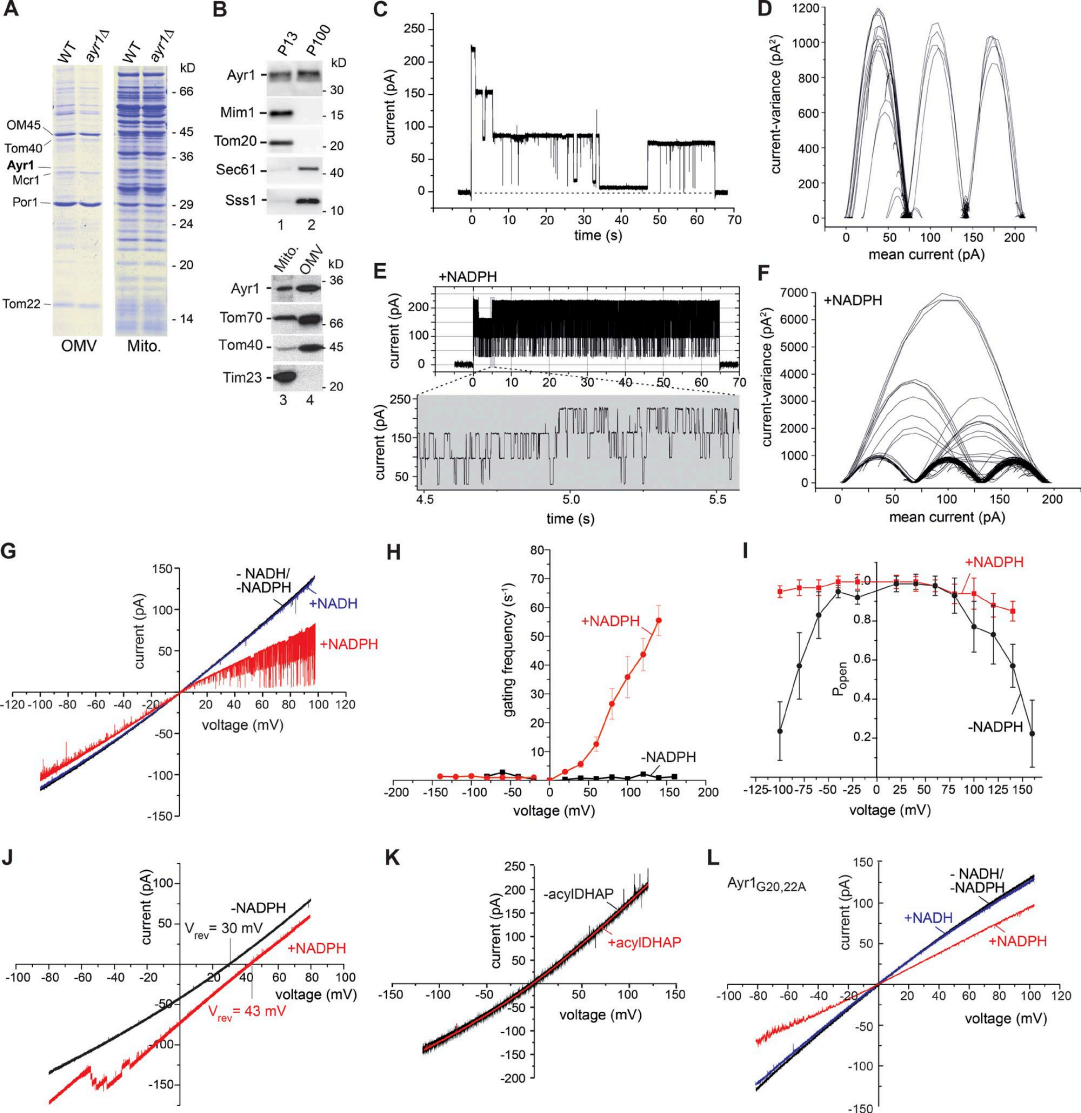
**Ayr1 forms an NADPH-regulated channel.** (A) Purified outer membrane vesicles (OMVs) and mitochondria (Mito.) from WT and *ayr1*Δ yeast were analyzed by SDS-PAGE and Coomassie staining. (B) Fractions enriched for mitochondria (P13) or microsomes (P100) and outer membrane vesicles were analyzed by SDS-PAGE and immunodetection. (C and E) Current recordings of the Ayr1 (404) channel in symmetrical (250 mM KCl cis/trans) buffer conditions at V_m_ = 140 mV in the absence (C) or presence (E) of 1 mM NADPH. (D and F) Mean variance plot of the Ayr1 channel in the absence (D) or presence of NADPH (F) at V_m_ = 140 mV. (G) Current-voltage recordings of the Ayr1 channel in symmetrical buffer conditions in the absence (black) or presence of 1 mM NADH (blue) or 1 mM NADPH (red). (H) Gating frequency of the Ayr1 channel in symmetrical buffer conditions in the absence (black) or presence of 1 mM NADPH (red). Mean values of three independent experiments with SD. (I) Open probability of the Ayr1 channel in symmetrical buffer conditions in the absence (black) or presence of 1 mM NADPH (red; *n* = 3). (J) Current-voltage recordings of the Ayr1 channel in asymmetric (250 mM/20 mM KCl cis/trans) buffer conditions in the absence (black) or presence of 1 mM NADPH (red). (K) Current-voltage recordings of the Ayr1 channel in symmetrical buffer conditions in the absence (black) or presence of 1 mM acyl-dihydroxyacetone-phosphate (acylDHAP; red). (L) Current-voltage recordings of the Ayr1_G20,22A_ channel in symmetrical buffer conditions in the absence (black) or presence of 1 mM NADH (blue) or 1 mM NADPH (red).

We found the 33-kD protein Ayr1 as an abundant protein in the mitochondrial outer membrane fraction (Fig. 4, A and B; Zahedi et al., 2006). Ayr1 was previously localized to the ER and lipid particles (Athenstaedt and Daum, 2000; Natter et al., 2005). The exact function of Ayr1 is unknown. It has been linked to various steps of lipid metabolism including reduction of 1-acyldihydroxyacetone-phosphate, triacylglycerol lipase activity, fatty acid elongation, and steroid biosynthesis as well as to cell wall biogenesis (Athenstaedt and Daum, 2000; Han et al., 2002; Vico et al., 2002; Ahn et al., 2010; Ploier et al., 2013). Upon cellular fractionation, Ayr1 was present in both mitochondrial and microsomal fractions (Figs. 4 B and S3, A and B). These results are consistent with a localization of Ayr1 in mitochondrial outer membrane and ER fractions, yet Ayr1 may also be linked to regions of close contact between ER and mitochondria (Vance, 2014; Herrera-Cruz and Simmen, 2017).

Purified and reconstituted Ayr1 showed channel activity in planar lipid bilayers with a main conductance of $\;{\bar{\Delta G}}_{max}≅1.47\;nS$ of the fully open channel as well as three smaller partially open states with conductance levels of $\bar{\Delta G}≅490\;pS\;$(Fig. 4, C and D). Ayr1 contains a predicted Rossmann fold (Geertz-Hansen et al., 2014) with a conserved nucleotide-binding motif (TGX_3_GXG) and further signature sequences characteristic for short-chain dehydrogenases (Vico et al., 2002; Oppermann et al., 2003; Kallberg et al., 2010). Short-chain dehydrogenases comprise a large protein family including NADPH-dependent oxidoreductases and aldo-keto reductases. Members of this protein superfamily are present in bacteria, archaea, and eukaryota (Kallberg et al., 2010). We asked whether Ayr1 was regulated by pyridine nucleotides (Kilfoil et al., 2013). NADPH strongly stimulated the frequency of gating between the distinct states (Fig. 4, E–H) and prevented the closure of the Ayr1 channel at high voltages (Fig. 4 I). Addition of NADPH led to an interconnection of the channel-open states (Fig. 4 F), demonstrating that the channel unit is formed by a single large pore comprising distinct smaller conductance states. In the absence of NADPH, the Ayr1 channel exhibited a reversal potential V_rev_ = 30 mV corresponding with a cation selectively of *P_K_^+^/P_Cl_^−^* = 4.5:1. NADPH led to an increased V_rev_ = 43 mV and a ratio *P_K_^+^/P_Cl_^−^* ≅ 10:1 (Fig. 4 J). Neither NADH nor the putative substrate 1-acyldihydroxyacetone-phosphate affected the channel properties of Ayr1 (Fig. 4, G and K). Upon replacement of glycines 20 and 22 of the NADPH binding motif of Ayr1 by alanines, the basic channel characteristics of Ayr1 were not altered; however, the effect of NAPDH was mostly abolished, and no increase of the gating frequency was observed (Fig. 4 L). We conclude that Ayr1 forms a cation-selective channel that is stimulated by NADPH.

### Conclusions

This study reveals an unexpected diversity of mitochondrial OMCs. The outer membrane fraction of yeast mitochondria contains twice as many channels as characterized to date (Table 1). The four channels known so far are β-barrel proteins. A systematic analysis of the yeast genome indicated that yeast contains no more than five membrane-integral β-barrel proteins (Imai et al., 2008; Kutik et al., 2008b; Zeth, 2010), the fifth one being the minor VDAC isoform Por2 that does not affect outer membrane permeability (Lee et al., 1998). The presence of eight channels thus implies that several of the new channels should be formed by α-helical membrane proteins. We identified two of the new channels on a molecular level: Mim1 and Ayr1. The MIM complex is largely formed by Mim1 proteins that contain a single predicted α-helical transmembrane segment (Popov-Čeleketić et al., 2008). We speculate that MIM is the first mitochondrial OMC formed by α-helical membrane proteins, though a high-resolution structure will be required to define its exact structure. Ayr1 contains a conserved Rossmann fold region characteristic of short-chain dehydrogenases (Vico et al., 2002; Oppermann et al., 2003; Kallberg and Persson, 2006) as well as a predicted α-helical hydrophobic segment (Athenstaedt and Daum, 2000). The Ayr1 channel is specifically stimulated by NADPH.

Our study leads to two major conclusions: (A) the number and diversity of mitochondrial OMCs are considerably larger than expected, challenging the classical model of the outer membrane as an unspecific molecular sieve, and (B) mitochondrial OMCs are likely not only formed by β-barrel proteins but also by proteins with predicted α-helical transmembrane segments.

## Materials and methods

### Yeast strains and growth conditions

The *Saccharomyces cerevisiae* strains and their corresponding genotypes are listed in Table S1. The yeast strains *por1*Δ (1281) and WT strains (1280, 1501, and 2624) have been described previously (Sikorski and Hieter, 1989; Blachly-Dyson et al., 1997; Sorger et al., 2004; Becker et al., 2011b). The *ayr1*Δ strain (1329) was generated by chromosomal integration of the *HIS3* marker in the *AYR1* locus via homologous recombination. Yeast strains were grown at 30°C in YPG (1% [wt/vol] yeast extract, 2% [wt/vol] peptone, and 3% [vol/vol] glycerol) or YPS (1% [wt/vol] yeast extract, 2% [wt/vol] peptone, and 2% [vol/vol] sucrose).

### Cellular fractionation, purification, and reconstitution of mitochondrial outer membrane vesicles into liposomes

Cellular fractionation and isolation of mitochondria were performed by differential centrifugation (Meisinger et al., 2006; Morgenstern et al., 2017). The mitochondrial membranes were separated after homogenization by sucrose density centrifugation (Zahedi et al., 2006). Attempts to fuse the purified outer membrane vesicles to the bilayer failed in >98% of the cases (≫50). In the few successful cases, the fusions produced a high number of channel activities in the artificial bilayer with very large currents up to the µA range. Therefore, we fused outer membrane vesicles with preformed liposomes, resulting in the incorporation of a lower number of different channel activities per single fusion event. With these mixed vesicles, fusion of the vesicles to the bilayer was successful in >95% of the cases. Liposomes were formed by subjecting a lipid mixture to sonication and three to four cycles of freeze-thawing. This lipid mixture (Avanti Polar Lipids) contained 20 mg/ml of lipids (46.5% l-α-phosphatidylcholine, 28.5% l-α-phosphatidylethanolamine, 9% l-α-phosphatidylinositol, 9% l-α-phosphatidylserine, and 7% cardiolipin; Kuwana et al., 2002) in 100 mM NaCl and 10 mM MOPS/Tris, pH 7.0. For reconstitution, outer membrane vesicles and preformed liposomes were solubilized using 40 mM or 80 mM Mega9, respectively. Subsequently, solubilized outer membrane vesicles and solubilized liposomes were mixed in a ratio of 1:4–10 (vol/vol) and incubated for 15 min at room temperature. The detergent Mega9 was removed by dialysis against 100 mM KCl and 10 mM MOPS/Tris, pH 7.0. For OMC7 and OMC8, *por1*Δ outer membrane vesicles were separated by blue native electrophoresis. The blue native gel was dissected into 20-gel slices, and proteins were electroeluted from the gel slices in the presence of *n*-heptyl-β-d-thioglucopyranoside (Meisinger et al., 2001; Becker et al., 2005). The eluted proteins were reconstituted into liposomes according to the approach described in this section. The resulting proteoliposomes were fused to planar lipid bilayers, and channel activities were determined by electrophysiology.

### Recombinant protein expression and purification

The proteins that were recombinantly expressed in this study are derived from the yeast *S. cerevisiae* and are listed in Table S2. The ORFs encoding the selected proteins were expressed with a His_10_ tag in *E. coli* BL21 (DE3) cells (Truscott et al., 2001). *E. coli* cells were grown in Luria–Bertani medium supplemented with ampicillin/chloramphenicol to an OD_600_ of 0.6–0.8 at 37°C. To induce recombinant protein production, IPTG was added to 1 mM final concentration. Protein expression was allowed for 3 h at 37°C. Subsequently, cells were isolated and shock frozen in liquid nitrogen. For purification of the expressed proteins, total cells were dissolved in 8 M urea, 100 mM NaP_i_, pH 8.0, 250 mM NaCl, and 20 mM imidazole for 60 min at 2°C. After a clarifying spin, the lysate was incubated with Ni-NTA–agarose (QIAGEN). Subsequently, the column material was washed with an excess amount of 6 M urea, 100 mM NaP_i_, pH 8.0, 500 mM NaCl, and 50 mM imidazole. Proteins were eluted with 6 M urea, 100 mM NaP_i_, pH 4.5, 10 mM Tris/HCl, and 500 mM imidazole. Mim1_His_ and Mim2_His_ were synthesized in wheat germ cell extract using a PCR-based template following the manufacturer’s recommendations (5 Prime). Proteins were purified under denaturing conditions (Ellenrieder et al., 2016).

### Generation of proteoliposomes containing expressed proteins

For reconstitution of recombinantly expressed proteins, preformed liposomes (46.5% l-α-phosphatidylcholine, 28.5% l-α-phosphatidylethanolamine, 9% l-α-phosphatidylinositol, 9% l-α-phosphatidylserine, and 7% cardiolipin) were solubilized with 80 mM Mega9, and the protein solutions were supplemented with 1% (wt/vol) SDS. Subsequently, liposomes and proteins were mixed in a protein/lipid ratio of 1:50 to 1:25 (wt/wt) and incubated for 15 min at room temperature to allow the formation of proteoliposomes. The lipid/protein mixture was dialyzed overnight at 4°C against 100 mM KCl and 10 mM MOPS/Tris, pH 7.0. In this step, Calbiosorb adsorbent (EMD Millipore) was added to remove the SDS. We optimized the lipid/protein ratio for each reconstitution. This is a crucial step to achieve single channel activity or activity of only few channels (*n* < 5) after fusion of the proteoliposomes with the planar lipid bilayer.

### Electrophysiological measurements

Electrophysiological characterization of proteins from mitochondrial outer membrane vesicles or of expressed and reconstituted proteins was performed using the planar lipid bilayer technique (Harsman et al., 2011; Krüger et al., 2012). For fusion with the planar lipid bilayer, proteoliposomes were added to the cis chamber in close proximity to the lipid bilayer. The fusion process was driven by osmotic pressure. Therefore, asymmetric buffer conditions were applied using 250 mM KCl and 10 mM MOPS/Tris, pH 7.0, in the cis chamber and 20 mM KCl and 10 mM MOPS/Tris, pH 7.0, in the trans chamber. We placed two Ag/AgCl electrodes linked by 2 M KCl-agar bridges into each chamber. The Ag/AgCl electrode in the trans chamber was connected to the head stage (CV-5-1GU) of a Geneclamp 500 current amplifier (Axon Instruments) and was used as the reference for exported membrane potentials. We used a Digidata 1200 A/D converter for current recordings. The obtained data were analyzed with a self-written program (“Ion channel Master;” Krüger et al., 2012). We adjusted the sampling interval to 50 µs for current recording. The signal was filtered with a low-pass filter at 5 kHz. All determined channel activities were controlled by independent means (Harsman et al., 2011). Independent experiments (*n* ≥ 3) were performed for the characterization of all channel activities. To exclude background signals, “empty” liposomes were included as mock samples.

### Analysis of proteins by immunodetection

Proteins were separated by SDS-PAGE. To assess the approximate molecular mass of proteins, we used Novex Sharp Prestain (Invitrogen) and SDS–Low Molecular Weight Marker (Sigma-Aldrich) as protein standards. For immunoblotting, polyvinylidene difluoride membranes were rinsed in methanol and soaked in transfer buffer (20 mM Tris, 150 mM glycine, 0.02% SDS, and 20% methanol). The SDS-PAGE gel was assembled in a semi-dry blotter on top of the polyvinylidene difluoride membrane in between Whatman paper preequilibrated with transfer buffer. The antibodies used and the incubation conditions for the immunoreactions are listed in Table S3. Primary and peroxidase-coupled secondary antibody incubations were performed in TBS buffer (20 mM Tris/HCl, pH 7.5, 125 mM NaCl, and 5% milk powder) for 1 h at room temperature. Affinity-purified antibodies were diluted in TBS buffer lacking milk powder. After excessive washing with TBS, enhanced chemiluminescence was used to detect the proteins using x-ray films or charge-coupled device cameras (LAS3000 or 4000; Fujifilm). The specificity of the antibodies was analyzed by comparing mitochondrial extracts from WT yeast cells, the corresponding yeast deletion strains, or mutant strains expressing the protein of interest with a tag. Upon separation by SDS-PAGE and immunoblotting, absence of the immunosignal in the corresponding deletion mutant or a size shift of the immunosignal in strains expressing the protein of interest with a tag confirmed the specificity of the antiserum.

### Online supplemental material

Fig. S1 shows statistical analysis of OMC7 and OMC8 channel activities related to Fig. 2. Fig. S2 shows control experiments to confirm the identity of the Mim1 channel and statistical analysis related to Fig. 3. Fig. S3 shows an analysis of cellular fractions and outer membrane vesicles related to Fig. 4. Table S1 provides a list of yeast strains and their corresponding genotypes. Table S2 provides a list of proteins that were recombinantly expressed. Table S3 provides a list of antibodies used in this study.

## Supplementary Material

Supplemental Materials (PDF)
