# Supplementary material for: Identification of new channels by systematic analysis of the mitochondrial outer membrane
Source: J Cell Biol. 2017 Nov 6;216(11):3485–95. doi: 10.1083/jcb.201706043 (PMC5674900; doi:10.1083/jcb.201706043)
Supplement: Supplemental Materials (PDF) [file JCB_201706043_sm.pdf]

Krüger et al., https://doi.org/10.1083/jcb.201706043

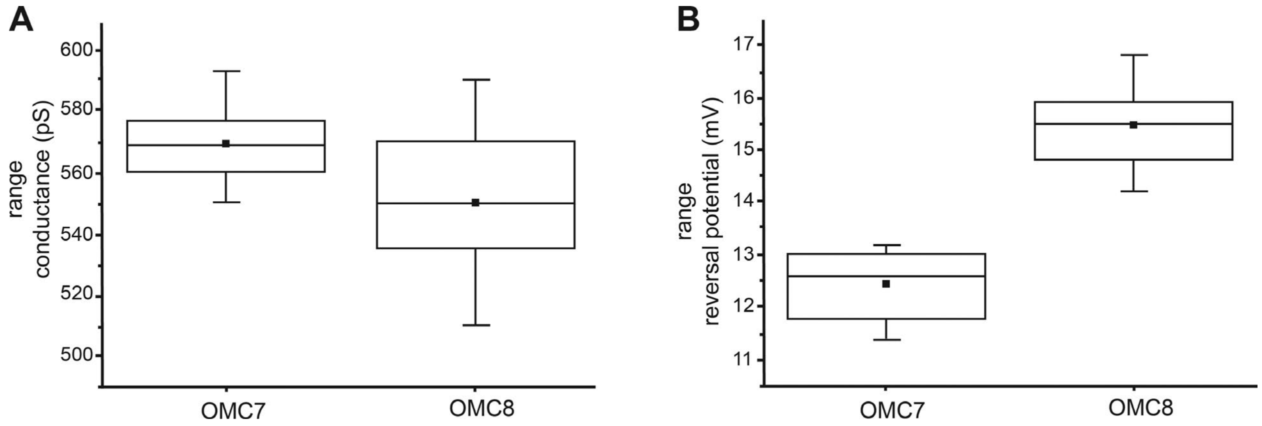

Figure S1. **Statistical analysis of conductance and reversal potential of OMC7 and OMC8.** (A) Box chart plots show the means (dots), medians (lines), 25th and 75th percentiles (bottom and top borders of the box), and minimum/maximum (whiskers) of the main conductance of OMC7 (n = 14) and OMC8 (n = 11). (B) Box chart plots of the reversal potential of OMC7 (n = 7) and OMC8 (n = 5).

THE JOURNAL OF CELL BIOLOGY

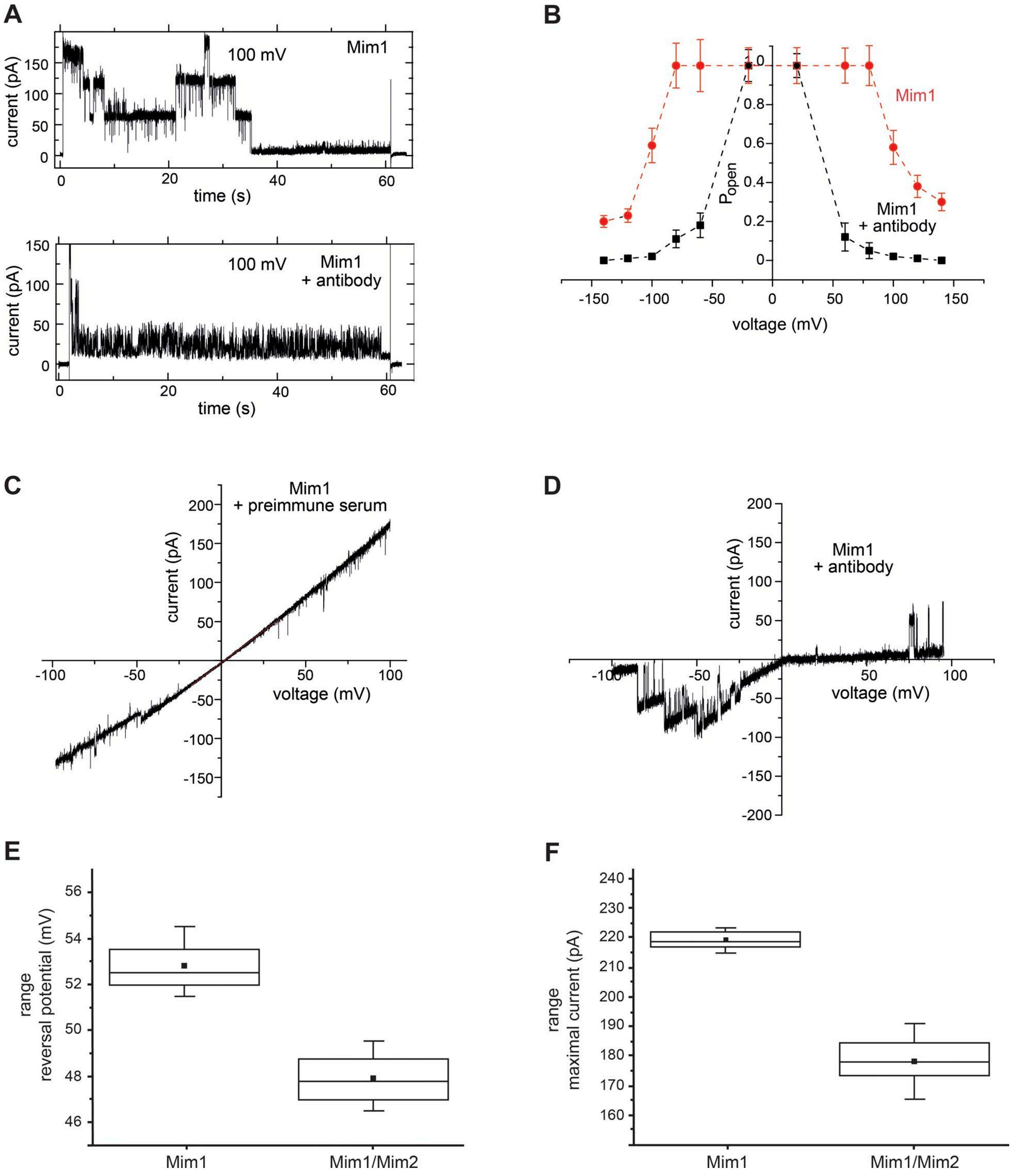

Figure S2. **Mim1-specific antibodies block the Mim1 channel.** (A) Current recordings of the Mim1 channel in the absence (top) or presence (bottom) of antibodies against Mim1. (B) Open probability of the Mim1 channel in the presence or absence of Mim1-specific antibodies. Depicted are mean values of three independent experiments with SD. (C and D) Current-voltage recordings of the Mim1 channel in the presence of preimmune serum (C) or Mim1-specific antibodies (D) under symmetrical buffer conditions. (E) Box chart plots show the means (dots), medians (lines), 25th and 75th percentiles (bottom and top borders of the box), and minimum/maximum (whiskers) of the reversal potential of Mim1 in the absence (*n* = 5) or presence of Mim2 (*n* = 4). (F) Box chart plot of the maximal current of Mim1 in the absence (*n* = 15) or presence (*n* = 11) of Mim2.

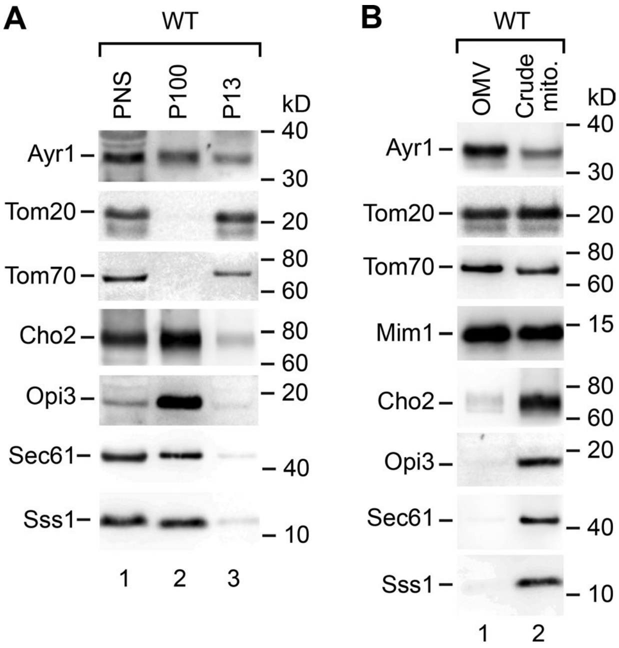

Figure S3. **Analysis of cellular fractions and mitochondrial outer membrane vesicles.** (A) The postnuclear supernatant (PNS) and fractions enriched for mitochondria (P13) or microsomes (P100) were analyzed by SDS-PAGE and immunodetection with the indicated antisera. (B) Highly purified mitochondrial outer membrane vesicles (OMVs) and a crude mitochondrial (Mito.) fraction including microsomal membranes were analyzed by SDS-PAGE and immunodetection with the indicated antisera. ER-localized proteins: Cho2, Opi3, Sec61, and Sss1.

Table S1. ***S. cerevisiae* strains used in this study**

**Strain**	**Genetic background**	**Number**	**Reference**
YPH499 (WT)	*MATa ura3-52 lys2-801_amber ade2-101_orchre trp1-Δ63 his3-Δ200 leu2-Δ1*	1,501	Sikorski and Hieter, 1989
W303 (WT)	*SUC2 GAL⁺ mal mel ade2-1 can1-100 his3-11,15 leu2-3,112 trp1-1 ura3-1*	2,624	Sorger et al., 2004
ayr1Δ	YPH499 *ayr1::HIS3*	1,329	This study
M3 (WT for *por1Δ*)	*MATa lys2 his4 trp1 ade2 leu2 ura3*	1,280	Blachly-Dyson et al., 1997
por1Δ	M3 *por1::LEU2*	1,281	Blachly-Dyson et al., 1997

Table S2. **List of constructs used for recombinant expression of proteins**

**Construct**	**Plasmid**	**Affinity tag**	**Source**
Om45	pET10C	no His-tag	This study
Scm4His	pET10C	C-terminal His tag	This study
Pth2His	pET10C	C-terminal His tag	This study
Mim1His	pET19b	N-terminal His tag	This study
Ayr1His	pET10N	N-terminal His tag	This study
Ayr1G20,22AHis	pET10N	N-terminal His tag	This study

Table S3. **List of antisera used in this study**

Antibodies directed against	Dilution	Number
S. cerevisiae Ayr1/404 (purified full-length protein)	1:100 TBS (affinity purified antibodies)	B 219
S. cerevisiae Mim1 (Cys-LKEISSPGTRGRVASKFL)	1:500 TBS + 5% milk	GR 544-1
S. cerevisiae Tom20 (purified protein comprising aa 32-183)	1:2,000 TBS + 5% milk	GR 3225-7
S. cerevisiae Tom22 (purified protein comprising aa 1-97)	1:2,000 TBS + 5% milk	GR 3227-2
S. cerevisiae Tom40 (purified full-length protein)	1:500 TBS + 5% milk	168-5
S. cerevisiae Tom70 (purified protein comprising aa 247-390)	1:500 TBS + 5% milk	GR 657-3
S. cerevisiae Tim23 (MSWLFGDKTPTDDAN-Cys)	1:500 TBS + 5% milk	133-4
S. cerevisiae Ssq1 (Cys-DNLIRQRLELISKADIMIS)	1:500 TBS + 5% milk	GR 1834
S. cerevisiae Sec61 (Cys-AKEGGFTKNLVPGFSDLM)	1:1,000 TBS + 5% milk	GR 760-7
S. cerevisiae Sss1 (Cys-EKLVEAPVEFVREGTQF)	1:500 TBS + 5% milk	GR 787-6
S. cerevisiae Cho2 (Cys-AHVISHRAWDIKQTLDSLA)	1:250 TBS + 5% milk	GR 5072-3
S. cerevisiae Opi3 (Ac-GIVRDMVYESALREQPTC)	1:250 TBS + 5% milk	GR 1392-2

References

Blachly-Dyson, E., J. Song, W.J. Wolfgang, M. Colombini, and M. Forte. 1997. Multicopy suppressors of phenotypes resulting from the absence of yeast VDAC encode a VDAC-like protein. *Mol. Cell. Biol.* 17:5727–5738. http://dx.doi.org/10.1128/MCB.17.10.5727

Sikorski, R.S., and P. Hieter. 1989. A system of shuttle vectors and yeast host strains designed for efficient manipulation of DNA in *Saccharomyces cerevisiae. Genetics.* 122:19–27.

Sorger, D., K. Athenstaedt, C. Hrastnik, and G. Daum. 2004. A yeast strain lacking lipid particles bears a defect in ergosterol formation. *J. Biol. Chem.* 279:31190–31196. http://dx.doi.org/10.1074/jbc.M403251200
